# Quantification of Human and Animal Viruses to Differentiate the Origin of the Fecal Contamination Present in Environmental Samples

**DOI:** 10.1155/2013/192089

**Published:** 2013-05-15

**Authors:** Sílvia Bofill-Mas, Marta Rusiñol, Xavier Fernandez-Cassi, Anna Carratalà, Ayalkibet Hundesa, Rosina Girones

**Affiliations:** Laboratory of Viruses Contaminants of Water and Food, Department of Microbiology, Faculty of Biology, Avenida Diagonal 643, 08028 Barcelona, Catalonia, Spain

## Abstract

Many different viruses are excreted by humans and animals and are frequently detected in fecal contaminated waters causing public health concerns. Classical bacterial indicator such as *E. coli* and enterococci could fail to predict the risk for waterborne pathogens such as viruses. Moreover, the presence and levels of bacterial indicators do not always correlate with the presence and concentration of viruses, especially when these indicators are present in low concentrations. Our research group has proposed new viral indicators and methodologies for determining the presence of fecal pollution in environmental samples as well as for tracing the origin of this fecal contamination (microbial source tracking). In this paper, we examine to what extent have these indicators been applied by the scientific community. Recently, quantitative assays for quantification of poultry and ovine viruses have also been described. Overall, quantification by qPCR of human adenoviruses and human polyomavirus JC, porcine adenoviruses, bovine polyomaviruses, chicken/turkey parvoviruses, and ovine polyomaviruses is suggested as a toolbox for the identification of human, porcine, bovine, poultry, and ovine fecal pollution in environmental samples.

## 1. Fecal Contamination of the Environment

Significant numbers of human microbial pathogens are present in urban sewage and may be considered environmental contaminants. Viruses, along with bacteria and protozoa in the intestine or in urine, are shed and transported through the sewer system. Although most pathogens can be removed by sewage treatment, many are discharged in the effluent and enter receiving waters. Point-source pollution enters the environment at distinct locations, through a direct route of discharge of treated or untreated sewage. Nonpoint sources of contamination are of significant concern with respect to the dissemination of pathogens and their indicators in the water systems. They are generally diffuse and intermittent and may be attributable to the run-off from urban and agricultural areas, leakage from sewers and septic systems, storm water, and sewer overflows [1–3].

Even in highly industrialized countries, viruses that infect humans prevail throughout the environment, causing public health concerns and leading to substantial economic losses. Many orally transmitted viruses produce subclinical infection and symptoms in only a small proportion of the population. However, some viruses may give rise to life-threatening conditions, such as acute hepatitis in adults, as well as severe gastroenteritis in small children and the elderly. The development of disease is related to the infective dose of the viral agent, the age, health, immunological and nutritional status of the infected individual (pregnancy, presence of other infections or diseases), and the availability of health care. Human pathogenic viruses in urban wastewater may potentially include human adenoviruses (HAdVs) and human polyomaviruses (HPyVs), which are detected in all geographical areas and throughout the year, and enteroviruses, noroviruses, rotaviruses, astroviruses, hepatitis A, and hepatitis E viruses, with variable prevalence in different geographical areas and/or periods of the year.

Moreover, with the venue of novel metagenomic techniques, new viruses are being discovered in the recent years that may be present in sewage and potentially contaminate the environment being transmitted to humans [4, 5]. 

Failures in controlling the quality of water used for drinking, irrigation, aquaculture, food processing, or recreational purposes have been associated to gastroenteritis and other diseases outbreaks in the population [6, 7]. Detailed knowledge about the contamination sources is needed for efficient and cost-effective management strategies to minimize fecal contamination in watersheds and foods, evaluation of the effectiveness of best management practices, and system and risk assessment as part of the water and food safety plans recommended by the World Health Organization [8, 9]. 

Microbial source tracking (MST) plays a very important role in enabling effective management and remediation strategies. MST includes a group of methodologies that aim to identify, and in some cases quantify, the dominant sources of fecal contamination in the environment and, more specifically, in water resources [10, 11]. Molecular techniques, specifically nucleic acid amplification procedures, provide sensitive, rapid, and quantitative analytical tools for studying specific pathogens, including new emergent strains and indicators. Quantitative PCR (qPCR) is used to evaluate the microbiological quality of water [12] and the efficiency of virus removal in drinking and wastewater treatment plants [13, 14] and as a quantitative MST tool [15].

Between a wide range of MST candidate tools (reviewed in [16–18]), the use of human and animal viruses analyzed by qPCR as fecal indicators and MST tools will be the focus of this review.

## 2. Indicators of Fecal Contamination

Fecal pollution is a primary health concern in the environment, in water, and in food. The use of index microorganisms (whose presence points to the possible occurrence of a similar pathogenic organism) and indicator microorganisms (whose presence represents a failure affecting the final product) to assess the microbiological quality of waters or food is well established and has been practiced for almost a century [19].

Classic microbiological indicators such as fecal coliforms, *Escherichia coli*, and enterococci are the indicators most commonly analyzed to evaluate the level of fecal contamination. However, whether these bacteria are suitable indicators of the occurrence and concentration of pathogens such as viruses and protozoa cysts has been questioned for the following reasons: (i) indicator bacteria are more sensitive to inactivation through treatment processes and by sunlight than viral or protozoan pathogens; (ii) nonexclusive fecal source; (iii) ability to multiply in some environments; (iv) inability to identify the source of fecal contamination; (v) and low correlation with the presence of pathogens.

Various authors concluded that these indicators could fail to predict the risk for waterborne pathogens including viruses [20, 21]. Moreover, the levels of bacterial indicators do not always correlate with the concentration of viruses, especially when these indicators are present in low concentrations [22, 23].

Those viruses that are transmitted via contaminated food or water are typically stable because they lack the lipid envelopes that render other viruses vulnerable to environmental agents. Moreover since viruses usually respond to a host specific behavior, their detection may provide data for MST.

The fact that rapid methods are required and that, moreover, many pathogens cannot be cultivated in the laboratory has led to the development of new methodologies for the study of pathogens and new proposed indicators of fecal contamination in water and food. These are based on the implementation of molecular techniques that are rapid and sensitive but may pick up both infectious and noninfectious (dead) types. Quantitative PCR assays are being considered by US-EPA as a rapid analytical tool [24]. A review focused on the application of qPCR in the detection of microorganisms in water has been recently published by Botes and coworkers [25].

## 3. Quantification of Human and Animal Viruses as a Tool-Box for Determining Presence and Origin of Fecal Contamination in Waters

The high stability of viruses in the environment, their host specificity, persistent infections, and high prevalence of some viral infections throughout the year strongly support the use of rapid cost-effective sensitive molecular techniques for the identification and quantification of DNA viruses which can be used as complementary indicators of fecal and urine (hereinafter “fecal”) contamination and as MST tools. Detection of excreted DNA viruses may allow the development of cost-effective protocols with more accurate quantification of contaminating sources compared to RNA viruses. This is due to the greater accuracy of qPCR and its/their lower sensitivity to inhibitors, as reverse transcriptase is not used when amplifying DNA viruses.

Our research group has proposed new viral parameters and methodologies for the detection and quantification of human and animal DNA viruses as fecal indicators as well as MST tools. The first viral markers proposed were DNA viruses such as human and animal adenoviruses and polyomaviruses, and the assays developed for their detection were based on qualitative PCR [22, 81–84], and more recently qPCR techniques have been developed for not only detecting but also quantifying these viruses in environmental samples [28–31, 37]. 

Several research groups are currently using these parameters for analysis of viral contamination in water and as MST tools. One of the objectives of this review is to examine available data, so far, on the application of specific DNA viral indicators proposed many years ago (human adenoviruses, JC polyomavirus, porcine adenovirus, and bovine polyomavirus) and to evaluate its usefulness as quantitative tools for determining the origin of the fecal contamination in different countries.

Our group recently developed quantitative PCR (qPCR) assays for the quantification of chicken/turkey parvoviruses and ovine polyomaviruses which, together with those previously proposed for human, bovine and porcine fecal contamination, might constitute a tool box for studying the presence and origin of fecal contamination in environmental samples (Table 1).

## 4. Treatment of Water Samples for Quantification of Viruses

A wide range of concentration methods have been described to recover viruses from water samples. These methods seek to concentrate viruses from large volumes (up to 1000 L) to smaller volumes ranging from 10 mL to 100 *μ*L. Most of the methods used are based on adsorption-elution processes using membranes, filters, or matrixes like glass wool [58, 62, 85]. However, they are two-step methods that can be cumbersome and could hamper the simultaneous processing of a large number of samples. In order to eliminate the bottleneck associated with two-step methods, and when volumes of 1–10 L are analyzed, a one-step concentration is used in our laboratory. The method was initially designed to concentrate viruses from seawater samples [38]. Briefly, the method is based on the addition of a preflocculated skimmed-milk solution to the volume of sample to be concentrated. The pH is then adjusted to 3.5 with HCl 1 N and the sample is then stirred for 8 h to allow the viruses to be adsorbed into the skimmed-milk flocs at room temperature (RT). Then flocs are recovered by centrifugation at 8,000 ×g for 30 min at 4°C. The supernatants are carefully removed without disturbing the sediment and the pellet is dissolved in phosphate buffer (pH 7.5). Preconditioning of the conductivity of the samples may be needed when applying the method to the concentration of viruses from freshwater samples [86] and a variation of the method has also been reported for sewage samples [87]. The method has proven to be efficient and reproducible, and by applying this method we have been able to concentrate virus from different water matrices [4, 52, 53, 77, 88, 89]. 

Enzymatic inhibition of the PCR is also a matter to have into consideration when testing environmental samples. Specific qPCR kits designed for working with environmental samples are available commercially. Analyzing neat but also diluted nucleic acids extraction is also recommended as well as introducing controls of inhibition in the assays performed [28].

Although some of these viruses, such as some types of human adenoviruses, may grow in cell culture, other viruses may not and/or cell culture assays take too long to produce rapid results. Some authors use nucleases treatment to destroy free genomes or genomes contained into damaged viral particles before nucleic acid extraction and qPCR in order to quantify only potentially infective viral particles [90–92]. 

**Figure 1:**
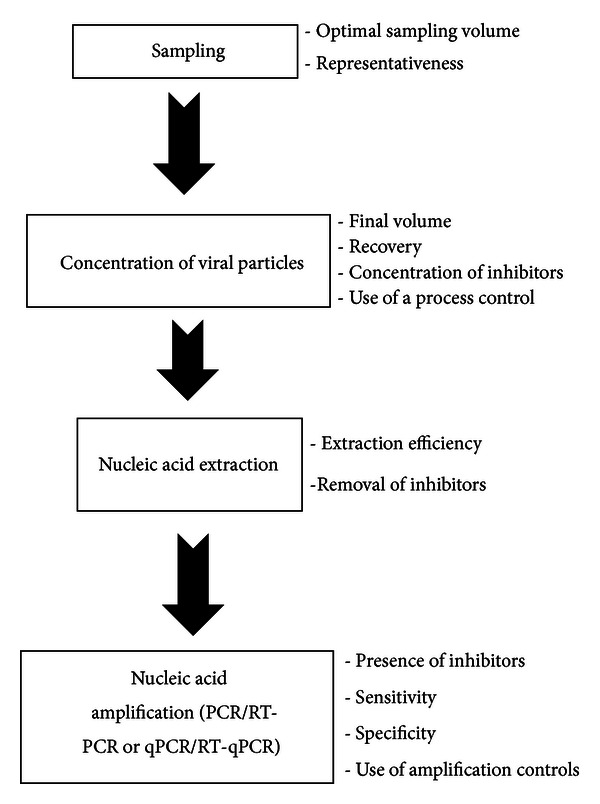
Flowchart of the method to detect and quantify viral indicators in the environment by PCR-based methods.

A flowchart summarizing the steps to follow to test an environmental sample for the presence of viral indicators is represented in Figure 1. Critical points to which attention should be paid are also summarized in the Figure 1.

## 5. Quantitative PCR of Human Adenoviruses and Human Polyomavirus JC: A Tool to Determine Human Fecal Pollution in Water Matrices

Some viruses, such as human polyomaviruses (HPyVs) and adenoviruses (HAdVs), infect humans during childhood, thereby establishing, some of them, persistent infections. They are excreted in high quantities in the feces or urine of a high percentage of individuals. 

The Adenoviridae family has a double-stranded DNA genome of approximately 35 000 base pairs (bp) surrounded by a 90–100 nm nonenveloped icosahedral shell with fiber-like projections from each vertex. Adenovirus infection may be caused by consumption of contaminated water or food, or by inhalation of aerosols from contaminated waters such as those used for recreational purposes. HAdV comprises 7 species with 52 types, which are responsible for both enteric illnesses and respiratory and eye infections [93].

Quantitative-based qPCR techniques used for the quantification of HAdV have been mainly designed to target the hexon protein and through degeneration of some nucleotides been able to amplify all HAdV types. In some cases and since HAdV types 40 and 41 are the ones etiologically associated to gastroenteritis as well as to a high prevalence in environmental samples, assays based on the sole detection of this two types have also been developed (Table 2).

Some of the more commonly used qPCR assays have been described by Hernroth et al. [26] with modifications [37] and Heim et al. [35]. We have previously compared both methods obtaining higher quantification in wastewater samples when applying the first one [37].

Table 2 summarizes quantitative HAdV data obtained by testing by qPCR different types of environmental samples.

Polyomaviruses are small and icosahedral viruses, with a circular double-stranded DNA genome of approximately 5000 bp that infect several species of vertebrates. JCPyV is ubiquitously distributed worldwide and antibodies against it are detected in over 80% of humans [94]. Kidney and bone marrow are sites of latent infection with JCPyV, which is excreted in the urine by healthy individuals [95, 96]. The pathogenicity of the virus is commonly associated with progressive multifocal leukoencephalopathy (PML) in immunocompromised states, and it has attracted new attention due to JCPyV reactivation and pathogenesis in some patients of autoimmune diseases under treatment with immunomodulators [97, 98]. JCPyV is ubiquitously distributed and antibodies against JC virus are detected in over 80% of human population worldwide. BKPyV, the other classical human polyomavirus, causes nephropathy in renal transplant recipients and other immunosuppressed individuals. It is also excreted in urine and thus is present in wastewater, although its prevalence is lower than that of JCPyV [82], JCPyV is more frequently excreted than BKPyV. Is for these reasons that the specific polyomaviral marker in use in our laboratory is based on the quantification of JCPyV [37]. The assay developed by McQuaig et al. [63] that targets JC and BK human polyomaviruses (HPyVs) has also been extensively used (Table 3). We have tested both assays in diverse types of environmental samples obtaining equivalent results (data not shown). Results obtained when applying these assays to environmental samples support the applicability of the proposed indicators as molecular markers of the microbiological quality of water and they would fulfill the conditions defined for a human fecal/urine indicator. Harwood et al. [65], in a study using PCR, suggest that human polyomaviruses were the most specific human marker for MST among many other tools analyzed. 

Overall, studies show that HAdV has the highest prevalence in environmental samples while JC polyomavirus (or HPyV) qPCR assays have the best specificity. For this reason we propose the analysis of both viruses, HAdV and JCPyV, to determine human fecal pollution of environmental samples (Table 1). It is important to point out that the proposed markers are selected for its stable excretion all over the year in all geographical areas. However, in some cases the numbers of specific pathogens in high excretion periods, such as rotaviruses or noroviruses, may exceed the numbers of HAdV [99].

## 6. Quantitative PCR of Animal Viruses: Determining Porcine, Bovine, Poultry, or Ovine Pollution Origin in Environmental Samples

Since porcine adenoviruses (PAdVs) and bovine polyomaviruses (BPyVs) were proposed as porcine and bovine fecal indicators [83, 84], several studies have shown that these viruses are widely disseminated in the swine and bovine population, respectively, although they do not produce clinically severe diseases (Table 4). 

In 2009 and 2010, quantitative assays for the quantification of these viruses were described to be applied to environmental samples [28, 29]. 

The results of these studies showed that BPyV and PAdV were quantified in a high percentage of the samples in which their presence was potentially expected, whereas samples used as negative templates were negative. BPyV and PAdV were found to be distributed in slaughterhouse wastewater and sludge, and in river water from farm-contaminated areas, but not in urban wastewater collected in areas without agricultural activities nor in hospital wastewater [28, 84, 100]. These results support the specificity and applicability of the BPyV and PAdV assays for tracing bovine and porcine fecal contamination in environmental samples, respectively. Quantitative data present in the literature on the presence of these viruses in environmental samples are summarized in Table 4. 

Recently, the quantification of chicken/turkey parvoviruses (Ch/TuPVs), highly prevalent in healthy chickens and turkey's from different geographical areas [101–103], has been reported as a candidate MST tool for the identification of poultry originated pollution in environmental samples [31]. A quantitative PCR assay targeting the Ch/TuPV VP1/VP2 region was developed (Table 1) and the viruses detected in 73% of pooled chicken stool samples from the different geographical areas tested (Spain, Greece, and Hungary). Also, chicken slaughterhouse raw wastewater samples and raw urban sewage samples downstream of the slaughterhouse tested positive. The specificity of the designed assays was further studied by testing a wide selection of animal samples (feline, canine, porcine, bovine, ovine, duck, and gull) as well as by testing hospital sewage and urban sewage from areas without poultry industry. These results indicate that Ch/TuPVs may be suitable viral indicators of poultry fecal contamination and that these viruses are being disseminated into the environment.

More recently, the quantification of ovine polyomavirus (OPyV), a newly described virus, has been reported as a candidate tool to identify an ovine fecal/urine origin of fecal pollution [30]. Putative OPyV DNA was amplified from ovine urine and faecal samples using a broad-spectrum nested PCR (nPCR) designed by Johne and coworkers [104]. A specific qPCR assay (Table 1) has been developed and applied to faecal and environmental samples, including sheep slurries, slaughterhouse wastewater effluents, urban sewage, and river water samples. Successful quantification of OPyV was achieved in sheep urine samples, sheep slaughterhouse wastewater, and downstream sewage effluents. The assay was specific and was negative in samples of human, bovine, goat, swine, and chicken origin. Ovine faecal pollution was detected in river water samples by applying the designed methods. These results provide a quantitative tool for the analysis of OPyV as a suitable viral indicator of sheep faecal contamination that may be present in the environment.

## 7. Conclusions

Specific qPCR assays for the quantification of DNA viruses have been proposed as specific and sensitive assays to quantify human, porcine, bovine polyomavirus, poultry, and ovine fecal contamination in environmental samples.

Quantitative data is being accumulated on the presence and concentration of the proposed viral markers in environmental samples in many different countries. Future efforts should be directed towards developing standard procedures and reference materials for a reproducible application of these tools.

Meanwhile, these assays can be used to evaluate the microbiological quality of water and the efficiency of pathogen removal in drinking and wastewater treatment plants and in MST studies.

## Figures and Tables

**Table 1:** Oligonucleotide primers and probes used for the detection and quantification of viral indicators.

Primers and probes	Virus	Position^a^	Reference	Sequence (5′-3′)
ADF	Human adenovirus (HAdV)	18869–18887		CWTACATGCACATCKCSGG
ADR		18919–18937	[26]	CRCGGGCRAAYTGCACCAG
ADP1		18889–18916		FAM-CCGGGCTCAGGTACTCCGAGGCGTCCT-BHQ1

JE3F	JC polyomavirus (JCPyV)	4317–4339		ATGTTTGCCAGTGATGATGAAAA
JE3R		4251–4277	[27]	GGAAAGTCTTTAGGGTCTTCTACCTTT
JE3P		4313–4482		FAM-AGGATCCCAACACTCTACCCCACCTAAAAAGA-BHQ1

QB-F1-1	Bovine polyomavirus (BPyV)	2122–2144		CTAGATCCTACCCTCAAGGGAAT
QB-R1-1		2177–2198	[28]	TTACTTGGATCTGGACACCAAC
QB-P1-2		2149–2174		FAM-GACAAAGATGGTGTGTATCCTGTTGA-BHQ1

Q-PAdV-F	Porcine adenovirus (PAdV)	20701–20718		AACGGCCGCTACTGCAAG
Q-PAdV-R		20751–20768	[29]	CACATCCAGGTGCCGC
Q-PAdV-P		20722–20737		FAM-AGCAGCAGGCTCTTGAGG-BHQ1

qOv_F	Ovine polyomavirus (OPyV)	VP1^b^ region		CAGCTGYAGACATTGTGG
qOv_R			[30]	TCCAATCTGGGCATAAGATT
qOv_P				FAM-ATGATTACCAAGCCAGACAGTGGG-BHQ1

Q-PaV-F	Chicken/turkey parvovirus (ChPV/TuPV)	3326–3345		AGTCCACGAGATTGGCAACA
Q-PaV-R		3388–3407	[31]	GCAGGTTAAAGATTTTCACG
Q-PaV-Pr		3356–3378		6FAM-AATTATTCGAGATGGCGCCCACG-BHQ1

^a^The sequence positions are referred to strains J01917.1 (HAdV), NC_001699.1 (JCPyV), D13942 (BPyV), AJ237815 (PAdV), and GU214706 (ChPV/TuPV) from Genbank. ^b^VP1: virion protein 1.

**Table 2:** HAdV quantification studies in environmental water matrices.

Authors [Reference]	qPCR detection method [Reference]	Matrices analyzed	Main results
He and Jiang, 2005 [32]	He and Jiang, 2005 [32]	Sewage and coastal waters	Mean values in sewage 8.1*E* + 05 GC/L. Serotypes 1–5, 9, 16, 17, 19, 21, 28, 37, 40, 41
Choi and Jiang, 2005 [33]	He and Jiang, 2005 [32]	River	2–4 logs GC/L, 16% positive samples
Haramoto et al., 2005 [34]	Heim et al., 2003 [35]	River	45% positive samples (29/64)
Albinana-Gimenez et al., 2006 [36]	Hernroth et al., 2002 [26]	River and sewage	River used as a source of water presented 4*E* + 02 GC/L
Bofill-Mas et al., 2006 [37]	Hernroth et al., 2002 [26]	Sewage, effluent, and biosolids	High HAdV quantities in sewage, effluent, and biosolids. *t*90 and *t*99 of 60.9 and 132.3 days
Calgua et al., 2008 [38]	Hernroth et al., 2002 [26]	Seawater	New skimmed-milk flocculation method to concentrate, mean values of 1.26*E* + 03 GC/L
Albinana-Gimenez et al., 2009 [39]	Hernroth et al., 2002 [26]	River and drinking-water treatment plants	90% positive for river water, mean values 1*E* + 01–1*E* + 04 GC/L
Dong et al., 2010 [40]	Heim et al., 2003 [35], and by Ko et al., 2005 [41]	Sewage, drinking water, and river and recreational waters	Adenovirus detected from all water types. 10/10 positives in sewage (1.87*E* + 03–4.6*E* + 06 GC/L), 5/6 positives in recreational waters (1.70*E* + 01–1.19*E* + 03 GC/L)
Hamza et al., 2009 [42]	Heim et al., 2003 [35]	River and sewage	97.5% positive river water samples (1.0*E* + 07–1.7*E* + 08 GC/L)
Ogorzaly et al., 2009 [43]	Hernroth et al., 2002 [26]	River	100% positive samples (1.0*E* + 04/l)
Bofill-Mas et al., 2010 [44]	Hernroth et al., 2002 [26]	Seawater	3.2*E* + 03 GC/L, HAdV41 the most prevalent
Haramoto et al., 2010 [45]	Ko et al., 2005 [41]	River water	HAdV more prevalent (61.1%) than JCPyV (11.1%)
Jurzik et al., 2010 [46]	Heim et al., 2003 [35]	Surface waters	96.3% positive samples (mean 2.9*E* + 03 GC/L and maximum of 7.3*E* + 05 GC/L)
Ogorzaly et al., 2010 [47]	Hernroth et al., 2002 [26]	Groundwater	HAdV was the most stable between MS2 and GA phages analyzed in groundwater
Rigotto et al., 2010 [48]	Hernroth et al., 2002 [26]	Seawater, lagoon brackish water, sewage, and drinking water	64.2% positive values (54/84)
Schlindwein et al., 2010 [49]	Hernroth et al., 2002 [26]	Sewage, effluent, and sludge	4.6*E* + 07–1.2*E* + 09 GC/L in sludge, 5*E* + 04–1.3*E* + 07 GC/L in sewage, and 3.1*E* + 05–5.4*E* + 05 GC/L in effluent
Aslan et al., 2011 [50]	Xagoraraki et al., 2007 [51]	Surface waters	2–4 logs GC/L, 36% positives (HAdV 40/41)
Calgua et al., 2011 [52]	Hernroth et al., 2002 [26]	Seawater	Mean values 1–3 logs GC/L
Guerrero-Latorre et al., 2011 [53]	Hernroth et al., 2002 [26]	River and groundwater	Low levels of HAdV in 4/16 groundwater samples
Hamza et al., 2011 [54]	Heim et al., 2003 [35]	River and sewage	3*E* + 03 GC/L in river and 1.0*E* + 07–1.7*E* + 08 GC/L in sewage
Kokkinos et al., 2011 [55]	Hernroth et al., 2002 [26]	Sewage	45.8% positive samples (22/48) in sewage. Main serotypes 8, 40, and 41
Souza et al., 2011 [56]	Hernroth et al., 2002 [26]	Seawater	HAdV as the most prevalent in seawater
Wong and Xagoraraki, 2011 [57]	Heim et al., 2003 [35]	Manure and sewage sludge	Concentrations of *E. coli* and *Enterococcus *correlate to HAdV (*P* ≥ 0.05) in sludge samples
Wyn-Jones et al., 2011 [58]	Hernroth et al., 2002 [26]	Recreational water	36.4% positive samples, more prevalent than noroviruses (9.4%)
Garcia et al., 2012 [59]	Hernroth et al., 2002 [26]	River (source water)	100% prevalence (1*E* + 07 GC/L)
Fongaro et al., 2012 [60]	Hernroth et al., 2002 [26]	Lagoon	96% positive samples (46/48)
Rodriguez-Manzano et al., 2012 [13]	Hernroth et al., 2002 [26]	Raw sewage, secondary and terciary effluents	100% positive samples for HAdV in all steps of the treatment. Removal of HAdV within primary and secondary treatments 1.03 log 10 (89%) and UV disinfection process 0.13 log 10 (11%)
Ye et al., 2012 [61]	Heim et al., 2003 [35]	River and drinking water	100% positive samples (24/24). Mean values in river 2.28*E* + 04 GC/L

**Table 3:** JCPyV (or HPyV) quantification studies in environmental water matrices.

Authors [Reference]	qPCR detection method [Reference]	Matrices analyzed	Main results
Albinana-Gimenez et al., 2006 [36]	Pal et al., 2006 [27]	Sewage and river	100% positive samples in sewage (5/5) and river (9/9). Mean values 2.6*E* + 06 and 2.7*E* + 01 GC/L, respectively
Bofill-Mas et al., 2006 [37]	Pal et al., 2006 [27]	Sewage, effluent, and sludge	99% positive samples. T99 of 127.3 days
Albinana-Gimenez et al., 2009 [62]	Pal et al., 2006 [27]	River	48% positive samples in river water
Albinana-Gimenez et al., 2009 [39]	Pal et al., 2006 [27]	River and drinking-water treatment plant (DWTP)	48% positive samples (different steps of the DWTP ) with mean values 1*E* + 01 to 1*E* + 03 GC/L
McQuaig et al., 2009 [63]	McQuaig et al., 2009 [63]	Sewage, fresh to marine water, animal waste	Mean values in sewage 3.0*E* + 07 GC/L
Hamza et al., 2009 [42]	Biel et al., 2000 [64]	River	Detected (as JC and BK) in 97.5% of the samples
Harwood et al., 2009 [65]	McQuaig et al., 2009 [63]	River, animal feces, and seawater	No detection of HPyV in animal feces No correlation with *Enterococcus* 100% host specificity
Ahmed et al., 2009 [66]	McQuaig et al., 2009 [63]	Sewage	
Abdelzaher et al., 2010 [67]	McQuaig et al., 2009 [63]	Seawater	The FIB levels exceeded regulatory guidelines during one event, and this was accompanied by detection of HPyVs and pathogens
Ahmed et al., 2010 [68]	McQuaig et al., 2009 [63]	Sewage and seawater	JC and BK are highly host-specific viruses and high titers are found in sewage
Bofill-Mas et al., 2010 [44]	Pal et al., 2006 [27]	River and sewage	Sewage ranges from 8.3*E* + 04 to 8.5*E* + 06 GC/L (7/7) River ranges from 4.4*E* + 03 to 1.4*E* + 04 GC/L (7/7)
Fumian et al., 2010 [69]	Pal et al., 2006 [27]	Sewage and effluent	JCPyV detected in 96% and 43% of raw and treated sewage, respectively
Haramoto et al., 2010 [45]	Pal et al., 2006 [27]	River	JCPyV prevalence 11.1%, BKPyV not detected
Jurzik et al., 2010 [46]	Biel et al., 2000 [64], and modified by Hamza et al., 2009 [42]	River	68.8% were positive for HPyV
Gibson et al., 2011 [70]	McQuaig et al., 2009 [63]	River and drinking water	HPyV were detected in one groundwater, three-surface water, and one drinking-water sample. No correlation with FIB
Hamza et al., 2011 [54]	Biel et al., 2000 [64]	River and sewage	River 5.0*E* + 01–3.8*E* + 04 GC/L, sewage 5.7*E* + 07–5.7*E* + 08 GC/L
Hellein et al., 2011 [71]	McQuaig et al., 2009 [63]	Seawater, sewage, and animal feces	Presence of HPyV in all sewage samples and in one freshwater sample
Kokkinos et al., 2011 [55]	McQuaig et al., 2009 [63]	Sewage	68.8% positive values (33/48) for JC and BK
Wong and Xagoraraki, 2011 [72]	McQuaig et al., 2009 [63]	Manure sewage and sludge	HPyV concentrations were slightly lower than *Escherichia coli* and *Enterococcus *(*P* < 0.05)
Chase et al., 2012 [73]	McQuaig et al., 2009 [63]	Recreational waters	HPyV detection near septic systems
Fongaro et al., 2012 [60]	McQuaig et al., 2009 [63]	Lagoon	21% positive samples
Gordon et al., 2013 [74]	McQuaig et al., 2009 [63]	Estuarine to marine waters and sewage spills	HPyV demonstrated the ability to detect domestic sewage contamination in water
Rodriguez-Manzano et al., 2012 [13]	Hernroth et al., 2002 [26]	Raw sewage, secondary and tertiary effluent	JCPyV in raw sewage (6/6) with an average concentration of 5.44*E* + 05 GC/L. Not detected in the tertiary effluent.
McQuaig et al., 2012 [75]	McQuaig et al., 2009 [63]	Seawater	Mean values 5*E* + 02 to 3.55*E* + 05 GC/L
Staley et al., 2012 [76]	Staley et al., 2012 [76]	Sewage, river	100% and 64% positive samples of sewage and river samples, respectively

**Table 4:** Quantification of PAdV and BPyV in environmental samples.

Authors [Reference]	qPCR detection method [Reference]	Matrices analyzed	Main results
Hundesa et al., 2009 [29]	PAdV, Hundesa et al., 2009 [29]	River, slaughterhouse, and urban sewage	100% positive samples in slaughterhouse sewage (1.56 + 03 GC/L) and 100% in river (8.38 GC/L)
Hundesa et al., 2010 [28]	BPyV, Hundesa et al., 2010 [28]	River, slaughterhouse, and urban sewage	91% positive samples in slaughterhouse sewage (2.95*E* + 03 GC/L) and 50% in river (3.06*E* + 02 GC/L)
Bofill-Mas et al., 2011 [77]	BPyV, Hundesa et al., 2010 [28]	Groundwater	1/4 well water positive for BPyV (7.74 × 10^2^ GC/L)
Wolf et al., 2010 [78]	PAdV, Wolf et al., 2010 [78]	River	50% positive river water samples
Wong and Xagoraraki, 2011 [57]	BPyV, Wong and Xagoraraki 2011 [57]	Sewage	100% positive for manure and wastewater, 5.6% positive for feces samples
Viancelli et al., 2012 [79]	PAdV, Hundesa et al., 2009 [29]	Manure	66% of the samples collected in the SMTS and in 78% of the samples collected in the DU system
Viancelli et al., 2013 [80]	PAdV, Hundesa et al., 2009 [29]	Manure	PAdV were more prevalent than other viruses and can possibly be considered as indicators of manure contamination
